# MicroVi: A Cost-Effective Microscopy Solution for Yeast Cell Detection and Count in Wine Value Chain

**DOI:** 10.3390/bios15010040

**Published:** 2025-01-12

**Authors:** Ismael Benito-Altamirano, Sergio Moreno, David M. Vaz-Romero, Anna Puig-Pujol, Gemma Roca-Domènech, Joan Canals, Anna Vilà, Joan Daniel Prades, Ángel Diéguez

**Affiliations:** 1 Department of Electronic and Biomedical Engineering, Universitat de Barcelona, Martí i Franquès 1, 08028 Barcelona, Spain; sergiomoreno@ub.edu (S.M.); canals@ub.edu (J.C.);; 2 eHealth Center, Faculty of Computer Science, Multimedia and Telecommunications, Universitat Oberta de Catalunya, Rambla del Poblenou, 156, Sant Martí, 08018 Barcelona, Spain; 3 INCAVI-IRTA, Catalan Institute of Vine and Wine—Institute of Agrifood Research and Technology, Plaça Àgora, 2, Vilafranca del Penedès, 08720 Barcelona, Spain; apuigpujol@gencat.cat (A.P.-P.);; 4 Institute of Semiconductor Technology (IHT) and Laboratory for Emerging Nanometrology (LENA), Technische Universität Braunschweig, Hans-Sommer Str. 66, D-38106 Braunschweig, Germany

**Keywords:** ab-on-a-chip, yeast cell count, holography, wine quality, chip-sized microscopy

## Abstract

In recent years, the wine industry has been researching how to improve wine quality along the production value chain. In this scenario, we present here a new tool, MicroVi, a cost-effective chip-sized microscopy solution to detect and count yeast cells in wine samples. We demonstrate that this novel microscopy setup is able to measure the same type of samples as an optical microscopy system, but with smaller size equipment and with automated cell count configuration. The technology relies on the top of state-of-the-art computer vision pipelines to post-process the images and count the cells. A typical pipeline consists of normalization, feature extraction (i.e., SIFT), image composition (to increase both resolution and scanning area), holographic reconstruction and particle count (i.e., Hough transform). MicroVi achieved a 2.19 µm resolution by properly resolving the G7.6 features from the USAF Resolving Power Test Target 1951. Additionally, we aimed for a successful calibration of cell counts for *Saccharomyces cerevisiae*. We compared our direct results with our current optical setup, achieving a linear calibration for measurements ranging from 0.5 to 50 million cells per milliliter. Furthermore, other yeast cells were qualitatively resolved with our MicroVi microscope, such as, *Brettanomyces bruxellensis*, or bacteria, like, *Lactobacillus plantarum*, thus confirming the system’s reliability for consistent microbial assessment.

## 1. Introduction

The wine industry is one of the most impactful industries in Europe, according to the annual report State of the World Vine and Wine Sector for 2023 [1]. Five European countries (Spain, France, Italy, Romania and Portugal) are present in the top ten countries regarding the vineyard surface area of the globe, netting a total up to the 40% of the vineyard surface area of the Earth agro-food field. In fact, Italy and Spain are the leading countries on research regarding the wine industry [2].

Focusing on quality in the value chain, wine quality has been an open research field in later years, and many authors have contributed to this field. For example, Petropoulos et al. proposed a solution to use fuzzy logic for wine quality estimation [3]. Other authors, like Morata et al., have studied the impact of yeast selection in the Iberian Peninsula during the last few decades, starting with isolation of the well-known *Saccharomyces cerevisiae* and other newly discovered yeast strains [4]. Recently, Swe et al. presented a work on how to use hyperspectral cameras to assess the quality of wine grapes using destructive and non-destructive methods [5]. Also, recently, Puig-Pujol et al. presented an application of ultra-high-pressure homogenization at different stages of wine production to enhance the quality of wine production by reducing the use of sulfites or other antimicrobial and antioxidant treatments [6].

In this work, we introduce a novel computer-assisted approach to address one of the critical open challenges in the wine industry by enhancing wine quality through the precise monitoring of the fermentation process. Specifically, this process hinges on accurately quantifying yeast cell concentrations during particular stages of wine production. This challenge is especially significant for sparkling wines, where stringent yeast counts are essential at specific production stages to ensure quality. While prior efforts have explored using smartphone cameras to assess certain wine quality parameters, our approach diverges by leveraging a microscope-on-a-chip solution composed entirely of cost-effective, off-the-shelf components. This setup, designed to function as a miniaturized laboratory, incorporates advanced computer vision techniques to offer a robust, automated alternative to traditional methods.

Our earlier research focused on the fundamental design and optimization of microscopes on a chip, as exemplified in our previous work. There, we demonstrated a lensless raster microscope based on a microdisplay, employing micro-LED arrays as illumination sources. This setup explored key optical parameters such as LED source characteristics, sample-to-display distances, and holographic correction techniques to enhance resolution [7,8]. However, the application scope of the earlier work was primarily confined to generalized biomedical assays without domain-specific adaptations. Here, we present a significant advancement by adapting this lensless microscopy concept to the unique requirements of the wine industry. Unlike the general-purpose configurations in prior studies, the proposed setup integrates microfluidic components tailored for handling wine samples, alongside a new software framework, MicroVi, which stands for microscopy-in-a-chip solution for wine samples, built in Python [9] with a QT-based graphical interface [10].

Moreover, with MicroVi application, we went with a setup built with full commercial hardware, focusing on reproducibility and stock options to ease distribution problems of these kinds of novel technological approaches, combining state-of-the-art advances with out-of-the-self devices, plus a solid software application. With this base design, we achieved a system capable of achieving a resolution of 2.19 µm, successfully resolving the G7.6 features from the USAF Resolving Power Test Target 1951, comparable to state-of-the-art optical microscopes. Thus, the results showed that MicroVi could accurately detect and count cells of Saccharomyces cerevisiae [6] and other contaminant yeast species, such as Brettanomyces bruxellensis, at concentrations relevant to the wine production process. The combination of cost-effective, off-the-shelf components and advanced image processing techniques positions MicroVi as a valuable tool for winemakers seeking an accessible yet highly precise solution for quality control in fermentation monitoring.

In summary, our contribution represents a domain-specific adaptation of the microscope-on-a-chip paradigm, integrating innovations in hardware and software to address a vital industrial need. This distinguishes MicroVi as a practical, accessible, and highly precise tool for winemakers, offering an unprecedented combination of cost efficiency and functionality in fermentation process monitoring.

## 2. Materials and Methods

### 2.1. Microscope on a Chip

Our device consisted of several components: a lensless camera board, a 3072 × 2048-pixel TIS-DMM-37UX287 (from The Imaging Source, Bremen, Germany; distributed by IberOptics, Madrid, Spain); a blue LED micro-display, the 640 × 480-pixel JBD013 Series (from Jade Bird Display, Shanghai, China); 62.5 µL microfluidic channels as laboratory disposable samples, µ-Slide I Luer Glass Bottom (from ibidi, Gräfelfing, Germany; distributed by Inycom, Zaragoza, Spain); and custom 3D-printed parts, printed in our facility using an Ultimaker 5 machine using PLA Black plastic (from Ultimaker, Utrecht, The Netherlands). The segmentation was performed with Ultimaker Cura, and the nozzle specifications were 0.4 mm, with the printing layer thickness set to 0.1 mm.

**Figure 1:**
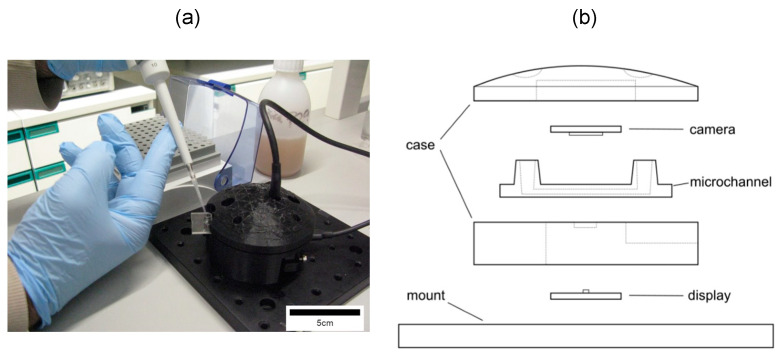
Our setup for the MicroVi solution to measure yeast cells in wine samples. (**a**) A photograph of the MicroVi while being used to prepare a sample of wine to be captured. (**b**) A schematic representation of MicroVi setup (not to scale), the setup is composed of a plastic case made with a commercial 3D-printed, a microfluidic channel which is a disposable element, a display that acts as the light source for this microscope, and finally, a digital CMOS camera.

Figure 1 depicts the mounted setup alongside a schematic representation of the parts of the setup. The microscope-on-a-chip setup has a *z*-axis mount. This is the propagation axis of the optical field, as is normal for such microscopes [7], and light is emitted from below the sample, from one or several LEDs from the microdisplay. Later, the light is scattered following in-line holography propagation and captured by the camera [11]. Table 1 shows a summary of the physical properties for the chosen components used to construct our microscopy-on-a-chip device; all of these properties are taken from the specifications that the different manufacturers provide for each of their respective components as the hardware is made from out-of-the-shelf components. Moreover, we added some relevant distances used in the setup (*z*-axis) relevant to the reconstruction of the holography images taken by the camera.

Note that MicroVi is device build from out-of-the-shelf components. This means that often certain parts of the microscope can be easily interchanged with other parts that are provided by the same manufacturer; for example, the microdisplay can be easily changed from one color to another (the manufacturer provides them with wavelengths centered in red, green and blue colors [12]), or the microchannel can be arrange to be interchanged with any of the provider’s channels that fit into the chamber (ibidi µ-Slide series), which is the same for the cameras (all DMx superior mounts).

### 2.2. Software and Graphic Interface

**Figure 2:**
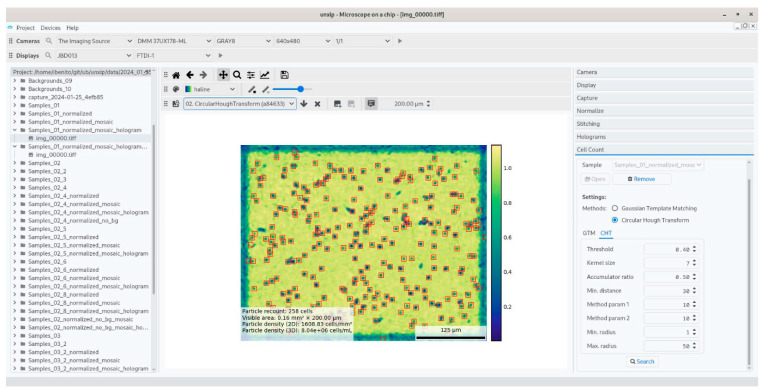
The MicroVi application is in use. The image shows the result of a holographic reconstruction performed with the MicroVi application. In this example, the application runs on a Linux device using Python and QT Frameworks.

We introduce a novel application to control the device, primarily developed in Python. Python was chosen, due to its versatile approach to different solutions, as a general-purpose language [9]. Python has access to modern computer vision libraries, and it can bind to popular graphic interface libraries, often written in C or other similar low-level languages, such as PySide2, open-source bindings to the QT framework [10]. The application views are designed using Qt Designer, and Figure 2 shows the application displaying the camera of the microscope open in a live stream. A summary of stock libraries that we used to implement our acquisition and computer vision pipeline is provided as follows:

(a) The PyData suite: NumPy, SciPy and matplotlib are the basic libraries for data science in Python, which are based in the abstraction of data arrays [13]. Also, the well-known pandas library was chosen to post-process the data created with the MicroVi application [14].
(b) Computer vision and machine learning frameworks: MicroVi implemented algorithms using OpenCV [15] and scikit-learn [16].
(c) Low-level image streaming: to interact with the lensless cameras, we built our application on top of the GStreamer [17] driver provided by the manufacturer.

### 2.3. In-Line Holography

**Figure 3:**
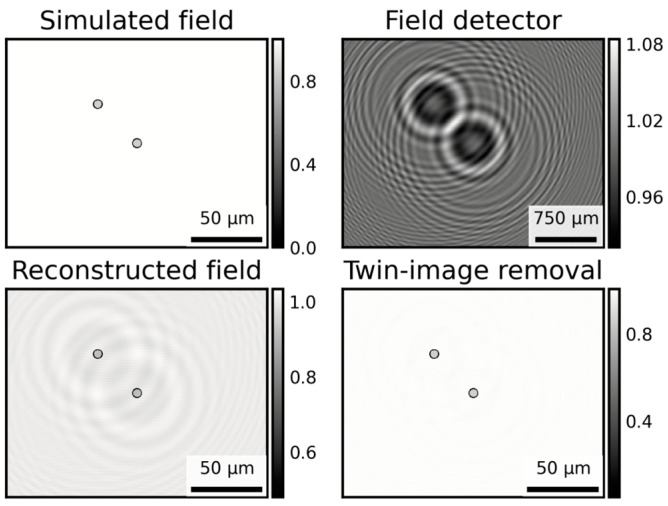
A simulation of an optical field (simulated field) with a sample of two particles with a non-transparent border (t = 0) and semi-transparent body (t = 0.15). The field is propagated up to the camera (field detector). The bar scale changes according to the projected in-line holography image size. The field is then reconstructed using a spherical wave propagator, assuming paraxial approximation (reconstructed field). Finally, an iterative (N = 5) phase recovery algorithm is applied to reduce the impact of the twin image (twin-image removal).

Lensless microscopy often involves some form of holography. We used the work previously introduced by Latychevskaia and Fink regarding in-line holography [11]. We modeled the light propagation in our microscope as spherical wave propagation in a paraxial approximation. First, we chose to use a spherical method as our wavefront originated from a single point, an LED from the microdisplay, and then, we checked that our mounted geometry fulfilled the paraxial approximation. In line with a previous experiment that took place, to simulate if this propagation, we followed the instructions below [11]:

(a) We created a simulated optical field t(x,y) for the image of two cells in the sample plane. These cells were simulated with a simple circle with zero-transmission function (t = 0) and a “body” of a certain transmission (t = 0.15), which can be seen as the “Simulated field” in Figure 3.
(b) We computed t(u,v), the Fourier transform of t(x,y).
(c) We simulated the propagator using the expression for spherical wave propagators $S(u,v)=exp(-i\pi \lambda z(u^{2}+v^{2}))$.
(d) We multiplied t(u,v) and S(u,v), and calculated the inverse Fourier transform of the result.
(e) We took the absolute value of the result to obtain $H_{0}(X,Y)$, which can be seen as the “Field detector” image in Figure 3.

After these steps, we proceeded to recover the image of the simulated field using the inverse process [11]:

(a) We computed $H_{0}(u,v)$, the inverse Fourier transform of $H_{0}(X,Y)$.
(b) We simulated the backwards propagator as $S^{*}(u,v)=exp(i\pi \lambda z(u^{2}+v^{2}))$.
(c) We multiplied $H_{0}(u,v)$ and $S^{*}(u,v)$ and calculated the Fourier transform of the result, which provides $\tilde{t}(x,y)$. The reconstructed field was derived from the holographic reconstruction method, which can be seen as the “Reconstructed field” in Figure 3.

In Figure 3, it can be seen that the reconstructed field displays several secondary waves in the recovered field. This is a common effect known as *twin-image* appearance. Optically, this twin image is originated by the fact that a “mirrored” version object is created when the algorithm is computed from twice the distance of the propagation (from the −z distance if z is the distance between the display and the camera). Mathematically, this is a problem of removing the phase of the complex field when computing $H_{0}(X,Y)$ in the simulation, as it takes the absolute value of the field to resemble the real scenario where our camera is not able to capture the phase of the holographic image.

(a) We followed an iterative approximation which consisted of retrieving the phase of the original field from a reconstructed image, which can be found elsewhere [18]. The method consists of propagating back and forth the field imposing physical constraints to the reconstruction. For our samples, we carried out the following:
(b) We recovered $\tilde{t}(x,y)$ using the back-propagation method, as fore-mentioned for recovering the field. And we split the complex field into amplitude and phase.
(c) We created a mask m(x,y) where the amplitude was higher than 1.
(d) We modified the amplitude to set to 1. The maxim allowed a physical value, the pixels in the mask. Plus, we set these pixels to 0 in the phase.
(e) We packed again the complex field and forward-propagated it to the capture plane, as fore-mentioned for simulating the propagation of the field to the camera.
(f) We split the new simulated field into amplitude and phase and took the phase and packed with the original amplitude $H_{0}(X,Y)$, creating a complex field, $H_{0}^{1}(X,Y)$, which is the first iteration of the twin-image removal algorithm.
(g) We repeated the steps N times from (a). The results for N = 5 can be seen in the “Twin-image removal” image in Figure 3.

### 2.4. Image Acquisition and Normalization

To address variability in LED intensity and minimize external light interference, a normalization technique was integrated into our image acquisition process. As opposite to the simulated holograms, for real-world holograms, the approach often taken for in-line holography involves capturing a background image with the same LED but without the sample [11,18]. This background image serves to normalize the sample image during reconstruction, ensuring independence from incident light variations. Mathematically, this process is expressed as follows:

$$H_{0}(X,Y)=\frac{H(X,Y)}{B(X,Y)}-1,$$

where $H_{0}(X,Y)$ is the normalized hologram, independent of the incident light, H(X,Y) is the hologram without normalization, and B(X,Y) is the background incident light. 

### 2.5. Multi-Holographic Mode and Mosaic Composition

Often, microscope-on-a-chip devices that incorporate a microdisplay can easily work in a “multi-holographic mode”. This is a feature that involves several pixels of the addressable array of LEDs. In this mode, several LEDs are used to capture the sample one by one. This produces one image per LED used this way. Often, the user can adjust the steps between LED usage, e.g., how many LEDs are skipped during capture [8].

Our computer vision pipeline included the implementation of the well-known SIFT method to extract features from the batch of images corresponding to the same sample; later, the features were matched with the BFMatcher algorithm [19]. Once the features are extracted and matched, the usual workflow in such scenarios is to fit a geometrical deformation model in order to better “stitch” the images to a certain “surface”, i.e., this is useful to avoid contributions of mismatched features. We preferred to fit an affine deformation by applying a least-squares bi-linear model—X and Y axes—instead of using a full perspective transformation as our stitching solution. Formulations for both approximations can be found in common pieces of literature [20].

**Figure 4:**
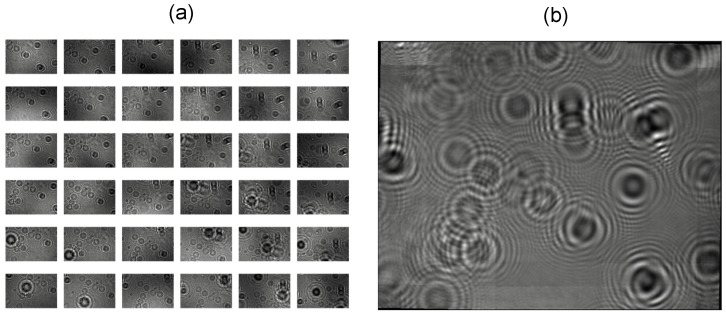
The stitching process of a sample image of *Brettanomyces bruxellensis* (CECT 1010) in our pipeline: (**a**) 64 images created changing the addressable LEDs from the microdisplay, and each image is normalized without a background image to enhance the different changes in the illumination; (**b**) the stitched image using SIFT, BFMatcher algorithms and a bi-linear model to solve the reconstruction of the sample image.

Figure 4 shows an example of this process. The mosaic is composed from up to 64 individual images. The image was generated from an actual sample of a contaminant yeast strain of *Brettanomyces bruxellensis* (CECT 1010)—not a simulation—and was normalized without a background—as we normally do follow the back-propagation method [18]—to show a clearer composition in the stitched image. It is interesting to notice that stitched images often introduce some border effects—e.g., areas of the mosaic that are defaulted to 0 in the border—and we mitigated this by implementing an apodization filter, a common technique used in combination with Fourier transforms introduced in the above subsection [21].

### 2.6. Cell Count

In the literature, the problem of counting cells has been tackled from a morphological point of view. As cells are approximately circular shapes, the gold-standard method to retrieve such shapes in the computer vision field is the circular Hough transform (CHT). We can find examples of this usage in agriculture or biomedical applications, for example: Alves et al. presented a method to use the CHT to detect honey comb cells from images [22]; Mahmood and Mansor presented a method to locate blood cells in optical microscopy [23]; and similar to our application, Pala and Yildiz presented a method to use the CHT using lensless imaging [24]. To adapt this methodology to our problem, we performed a grid search fine-tuning of the parameters of the CHT algorithm over the control samples that were also measured manually. This can be seen as a way to “train” the CHT algorithm for our images [25].

### 2.7. Sample Preparation

In order to present qualitative and quantitative evidence that our microscope-on-a-chip solution worked as intended, we targeted *Saccharomyces cerevisiae* species, which is specifically intended for oenological use [4]. For *S. cerevisiae*, we studied the strain P29 (CETC 11700, from Spanish Type Culture Collection, València, Spain) which is a yeast strain widely used in sparkling wine elaboration. It was isolated from the Appellation of Origin Penedès (Barcelona, Spain). Plus, we also targeted some spoilage species, i.e., *Brettanomyces bruxellensis* strains: one from stock (CECT 1010, from Spanish Type Culture Collection, València, Spain) and *B. bruxellensis* wild yeast, which was part of the INCAVI yeast collection that was isolated from a red wine from the Penedès region. Moreover, we also tested our microscopical setup with a commercial lactic acid bacteria species for oenological use: *Lactobacillus plantarum* (strain ML Prime™ from Lallemand Inc., Montreal, QC, Canada).

For *S. cerevisiae* samples, yeast starter culture samples were inoculated in pasteurized must and incubated for 48 h at 28 °C until reaching a concentration ranging between 100 and 150 million cells per milliliter. This was carried out twice, first, in order to calibrate the measures from the MicroVi prototype against an optical microscope; and second, to study the free evolution of yeast concentration on a sample during the fermentation process. Subsequently, for the first scenario, serial dilutions of the yeast culture were conducted to ascertain the optimal yeast count range for the MicroVi prototype. Once the optimal range for yeast counting with the prototype was determined, the evolution of yeast concentration during the fermentation process was studied. For this, serial dilutions were performed as in the first case.

Moreover, yeast concentration during the tirage stage of sparkling wine production was assessed. To accomplish this, the initial inoculation of sparkling wine samples was conducted, with yeast concentrations ranging between 1 and 2 million cells/mL, followed by an evaluation. Regarding commercial bottled wines, two of them were subjected to an evaluation to ascertain the absence of yeast.

Finally, all samples were introduced in the chip-sized microscope using a 62.5 μL microfluidic channel (µ-Slide I Luer Glass Bottom). After depositing the samples into the microfluidic channel, we waited 5 min for the samples to settle into a layer of cells at the bottom of the channel.

## 3. Results

### 3.1. Holography Reconstruction and Optical Resolution

**Figure 5:**
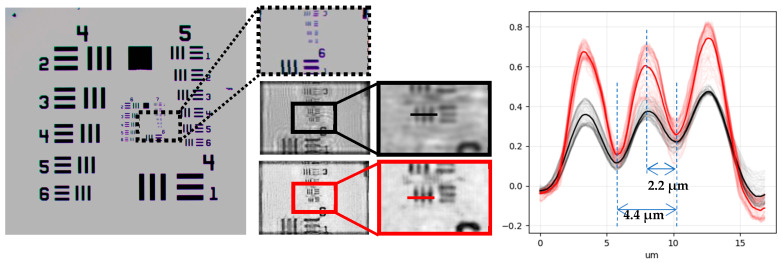
The USAF Resolving Power Test Target 1951 is captured, first, with an optical microscope (**left** image). Then, the ROI region corresponding the smaller targets from the 7th group are zoomed in (dashed lines, **top** image). Also, the same region is captured using the MicroVi device: the image is recovered using the holographic method (**middle** image) and later zoomed in (black lines); the image is cleaned using the twin-image removal method (**bottom** image) and later zoomed in (red lines). Finally, a mean profile of the USAF target for the smallest distance in group seven is shown in the plot. Once measured, the cleaned version increases the signal of the pattern by two. The measures show the recovered period values (2.2 µm and 4.4 µm) corresponding to the G7.6 pattern from the USAF Resolving Power Test Target 1951.

The resolution of the MicroVi device was determined by resolving the well-known USAF Resolving Power Test Target 1951 (see Figure 5). This target is a known reference pattern used by many others in research also related to imaging devices for agricultural applications [26,27]. The target comprises a series of repeating squared patterns called “groups”. Within each group, a descending amplitude pattern is depicted. With our MicroVi software, we captured an array of 64 images and stitched the image into a mosaic image (similar to Figure 4). Then, we applied a holographic back-propagation to the target plane and implemented the iterative phase-removal algorithm to clean the twin-image noise (similar to Figure 3). The results can be observed in Figure 5. MicroVi was able to qualitatively resolve the sixth pattern of the seventh group from the USAF 1951 (G7.6), which was the smallest feature in our target. Quantitatively, we measured a half-period of the pattern of 2.2 ± 0.1 µm, which was consistent with the tabulated measure of the G7.6 pattern, 2.19 µm.

**Figure 6:**
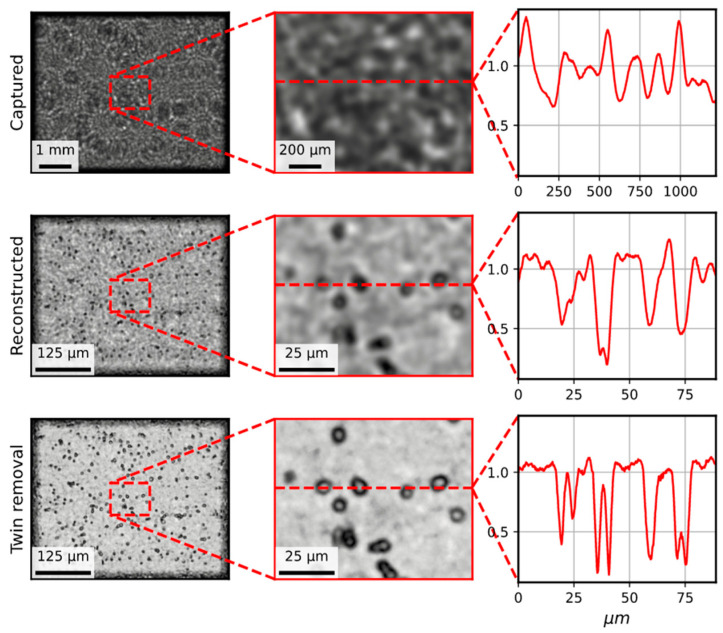
A sample of *Saccharomyces cerevisiae* species. The first row shows a stitched image from 64 captures using the MicroVi microscope; a central ROI of the image is then focused and a profile is extracted; the profile is evidently meaningless. The second row shows the holographic reconstruction of the stitched image; the same ROI is extracted, and the same profile is shown; and then, the yeast cells start to emerge. Finally, the third row shows the twin-image removal method, which qualitatively increases the resolution of the image; the cells start to be resolved.

Figure 5 also shows the importance of the twin-image removal algorithm, which increases the contrast between the background of the image (higher values in the color profile graph) and the important features (lower values). Plus, Figure 6 shows evidence on the key role the twin-image removal algorithm in order to properly resolve cells of a *S. cerevisiae* species sample. The color profiles across the ROI of the given cells show how the cell can be resolved as an entity only using a one-step holographic reconstruction, but its morphology becomes more explicit after the image is cleared with the method.

### 3.2. Qualitative Results on Different Species and Wines

**Figure 7:**
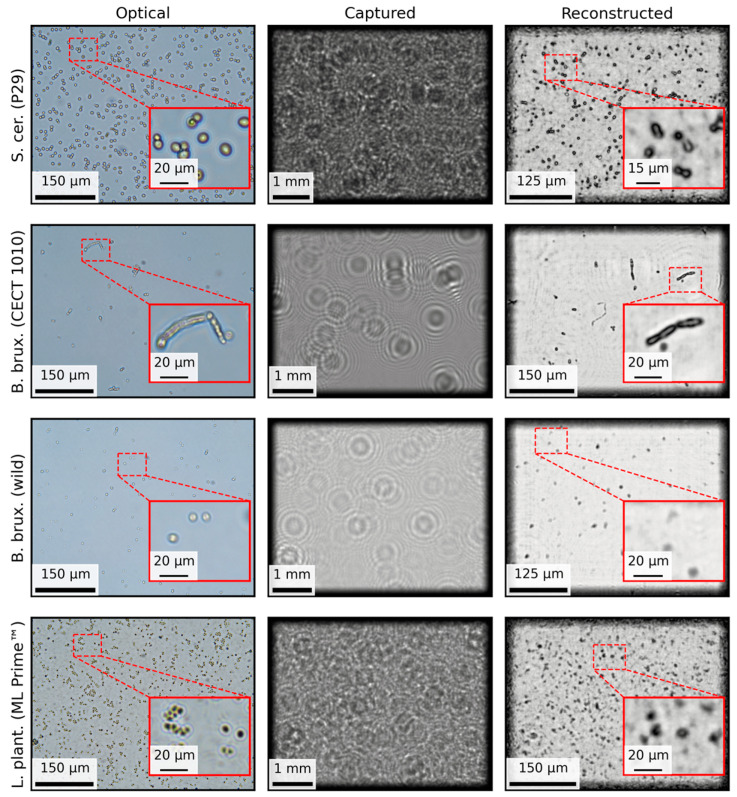
A comparison for different species of yeast cells and lactic acid bacteria. The columns show the same three steps for each species: optical, an image of a sample captured with the optical microscope; captured, the same sample—but not the same spot—captured with the MicroVi microscope, and images are shown as diffraction patterns; reconstructed, holographic reconstructions plus the twin-image removal algorithm. The rows show four different species, three yeast species, the last two contaminant species, and *Lactobacillus* species, namely, *Saccharomyces cerevisiae* P29 (CECT 11770 strain), *Brettanomyces bruxellensis* (CECT 1010 strain), *Brettanomyces bruxellensis* (wild strain), and *Lactobacillus plantarum* (ML Prime™ strain).

On the one hand, we tested our microscope under a variety of yeast strains and lactic acid bacteria (LAB). Figure 7 illustrates the performance of the MicroVi system in detecting different species of yeast cells and LAB, comparing results from traditional optical microscopy, diffraction pattern images captured with MicroVi, and the final holographically reconstructed images. Both S. cerevisiae (P29 strain) and B. bruxellensis (CECT 1010 strain) were successfully resolved, showing clear structural details of the cells.

These details are important for yeast morphology analysis and demonstrate MicroVi’s ability to recover shape and form. However, the wild strain of *B. bruxellensis* presents challenges due to its smaller size, resulting in a less distinct reconstruction, where the cell shapes are not as clearly defined as with the stock strains. Despite this, the morphology is different enough to assess the differences qualitatively and detect them as a contaminant species. Additionally, *Lactobacillus plantarum* (ML Prime™ strain) cannot be resolved at the individual cell level. Instead, clusters of *Lactobacillus* appear as merged entities in the reconstructed images, suggesting that the current resolution of MicroVi is insufficient to differentiate these closely packed bacterial cells.

**Figure 8:**
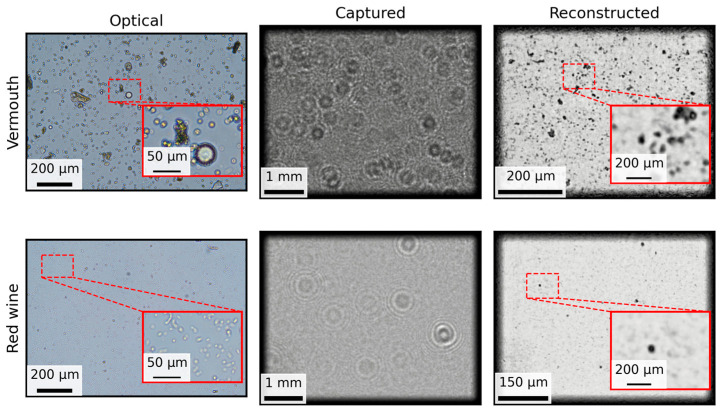
A comparison of the use of MicroVi microscopy for two different commercial samples. The columns show the same three steps for each species: optical, an image of a sample captured with the optical microscope; captured, the same sample—but not the same spot—captured with the MicroVi microscope (the images are shown as diffraction patterns); reconstructed, holographic reconstructions plus the twin-image removal algorithm. The rows show two different commercial samples from vermouth and red wine.

On the other hand, Figure 8 presents a comparison of MicroVi microscopy applied to two different commercial samples: vermouth and red wine. These tests were conducted to simulate real-world scenarios where wine samples, rather than laboratory-prepared cultures, are analyzed. In the vermouth sample, a variety of debris from different materials in the liquor, such as spices, was observed. These particles were clearly distinguishable from the yeast cells present in the sample. The microbiological count for this sample was revealed to be 390 cells per milliliter after filtering 300 mL of the sample and concentrating the retained material in 1 mL of sterile water. This high yeast count, along with the presence of other debris, was corroborated by the microscopy results.

On the other hand, the red wine sample exhibited smaller yeast cells that could not be resolved clearly by MicroVi, indicating the limitations of the system for detecting smaller microorganisms in complex samples. The microbiological analysis of this red wine sample showed 20 cells/mL after filtering 200 mL of wine and concentrating it in 1 mL of water. Despite the low yeast count, the results demonstrate that MicroVi can still capture the presence of small amounts of yeast, although with limited resolution. Additionally, the red wine coloration posed no problem for the MicroVi device, further supporting its potential applicability in real-world conditions.

### 3.3. Cell Counts and Device Calibration

**Figure 9:**
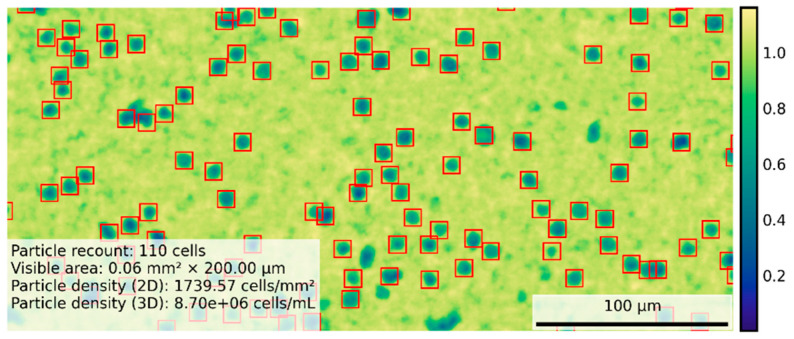
A view of the MicroVi program, which displays a reconstructed image ROI from *S. cerevisae* (P29 strain) yeast cells in artificial wine. The image depicts the captured image with a color scheme where yellowish colors are the background of the sample, and the blue patterns are the recovered cells. The image also displays a recount legend, with bounded boxes of the found cells.

To ensure our setup was able to count yeast cells in the same fashion that a human can count cells from our lab optical microscope (Nikon Eclipse Ci), we performed a simple experiment to assess the regression calibration between the two methods. In order to count the cells, we used the MicroVi framework to capture different labeled concentrations of *S. cerevisiae* cells (strain P29, CECT 11700), as described before. We diluted the *S. cerevisiae* culture samples and performed a count in both setups. Comparing the results, we established that the optimal yeast count range of the MicroVi prototype for our application should be between 0.5 and 50 million cells per milliliter. Figure 9 depicts a result of the MicroVi software counting cells using the Circle Hough Transform method, displaying an example of around 8 million cells per milliliter.

**Figure 10:**
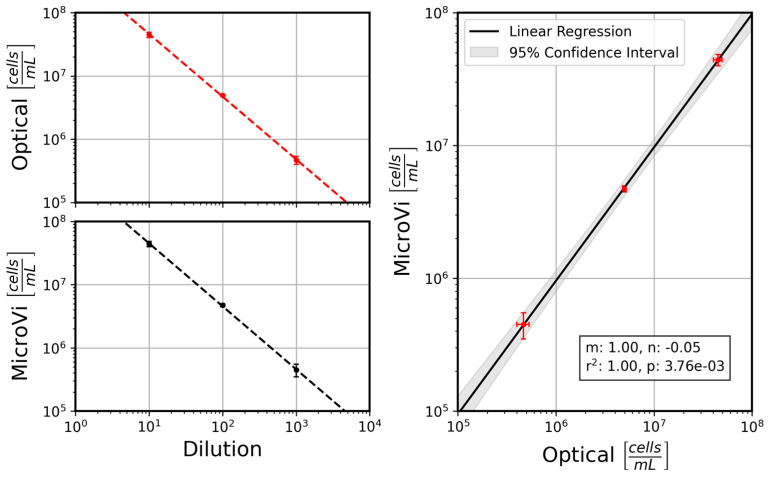
A comparison between the optical microscope and MicroVi (lab-on-a-chip) cell count measurements across dilutions for *S. cerevisae* (P29 strain) samples. The left column shows the cell count as a function of dilution for both the optical microscope (**top**) and MicroVi (**bottom**). The right column shows the linear regression between the two measurement techniques, with MicroVi closely aligning with the optical measurements.

In terms of calibration, Figure 10 displays the comparative performance between the optical microscope and the MicroVi software for each dilution level. The left-side plots show the performance of each counting method against the dilution, showing good agreement between the two methods, with both sets of data exhibiting a clear linear relationship across multiple dilutions. The right-side plot highlights the linear regression analysis, indicating a near-perfect correlation between the MicroVi counts and the manually obtained counts from the optical microscope. The slope of the linear regression line is close to 1, with a negligible intercept (*m* = 1.00, *n* = −0.05), confirming that MicroVi can replicate the results from the optical microscope with high accuracy. This minimal but consistent intercept shows that MicroVi technology is under-counting the cells; however, this is not important as decades of cells are clearly separable from each other. In other terms, the false negatives in recount do not exceed the proper margins established from the application for wine yeast cell counts. And this can also be qualitatively appreciated in Figure 9. Mainly, the not-counted cells are corner cases of the reconstruction algorithm (i.e., out of focus, etc.).

These results suggest that the MicroVi system can serve as a reliable tool for automated cell counting in a lab environment for the target range (0.5–50 million cells per milliliter), with its performance closely matching that of manual methods using optical microscopy. The confidence intervals, as shown in the regression plot, further support this conclusion, demonstrating the system’s robustness and potential to standardize cell counting across different operators and experimental conditions.

## 4. Discussion

The pipeline used for cell counting in the MicroVi system leverages advanced computer vision techniques to ensure accurate and reliable results for quality microscopy for the agriculture vineyard industry. First, the system captures multiple holographic images, which are then stitched together to form a composite image using feature matching algorithms like SIFT [19]. Following this, a Circular Hough Transform (CHT) is employed to detect the circular yeast cells, as described in similar work for cell detection in biomedical applications [23]. This approach ensures that the system can identify yeast cells with high precision, making it suitable for applications in the sparkling wine industry as yeast growth is crucial in its fermentation process [28].

One potential improvement to the current pipeline lies in the way the individual images from the multi-holographic reconstruction are stitched. At present, the SIFT algorithm is used to obtain vectors that describe the displacements between images, and an affine model is applied to fit these displacements. This method assumes that all images are displaced in a uniform manner. However, there are several alternatives that could enhance the accuracy of the stitching process. For example, employing a projective homography deformation fitting [29], as implemented in the OpenCV framework, could account for more complex image displacements—e.g., optical aberrations. Furthermore, thin-plate spline (TPS) approximation offers another solution as this technique has been successfully applied in various fields [30]. By using TPS, the system could better handle local distortions in the images, resulting in a more accurate reconstruction of challenging fittings.

Another area for improvement lies in the order of the stitching and holographic reconstruction steps. Currently, the images are stitched before the holographic reconstruction is performed. However, an alternative approach would be to reconstruct holograms of the individual images first and then stitch them afterward. This would allow for depth-resolved reconstructions through multilayer deconvolution [31], which could help mitigate false negatives in the cell counting process. By reconstructing multiple layers, the system could capture cells that might otherwise be missed due to overlapping structures in a single plane. Additionally, taking advantage of the distribution of displacement vectors obtained from the SIFT detector could enable layer-specific stitching. Each layer could be aligned and stitched separately, ensuring that cells at different depths are accurately reconstructed and counted. This multilayer approach could significantly enhance the robustness of the cell count in samples with complex structures, such as those found in wine samples with multiple yeast or bacterial species. Or more simply, this could enable registering cells without having to wait until they deposit in the inferior layer, performing a dynamic measurement of the sample.

Regarding cell count, an alternative to the CHT method is the well-established YOLO deep learning architecture, which has demonstrated promising results in the detection and counting of other types of cells—e.g., blood cells [32]. Its implementation within the MicroVi pipeline would require its application to the reconstructed holographic images, where it could potentially detect yeast cells with greater speed and accuracy than CHT. By using YOLO, the system could not only enhance its real-time performance but also reduce the complexity of manually tuning the detection parameters. Despite this, the main issue with YOLO-based architectures is that they need expensive training and dataset labeling.

On the other hand, recent research has shown the potential of using deep learning encoders to label-free recover features from raw diffraction patterns, eliminating the need for a separate holographic reconstruction step [33]. These approaches employ deep fully convolutional neural networks (FCNNs) to detect and count cells directly from diffraction patterns, significantly simplifying the image processing pipeline. Similarly, other authors have demonstrated that deep learning models can infer cell positions in lens-free microscopy images without traditional reconstruction methods [34]. These findings suggest that integrating a deep learning-based approach into the MicroVi framework could streamline the process, making it more efficient while maintaining or even improving accuracy in cell detection [35].

By utilizing these deep learning techniques, the MicroVi system could evolve into a more robust and scalable solution for yeast cell detection, offering improved automation and adaptability. The ability to bypass traditional image reconstruction processes opens up new possibilities for enhancing the system’s overall performance, particularly in complex samples where overlapping or indistinct cell structures might otherwise pose challenges for conventional methods.

Finally, beyond our current setup, which implies a static measurement of the sparkling wine samples, e.g., a snapshot of the current concentration of yeast cells, enabling a dynamic measurement of the cell concentration by the use of micro-pumps [36] that could help circulate the wine through the MicroVi setup could enable an increase in the dynamic range of the device in terms of cell count. This is possibly because such fluidic circuits can dilute the samples into the current dynamic range of the device, or this is due the fact that we can circulate cell particles over time and compute an average measurement, increasing the apparent search area. Both techniques with such circuits would benefit both the inferior and superior detection limits.

## 5. Conclusions

The development of MicroVi marks an advancement in the application of chip-sized microscope technology for the wine industry. By utilizing commercially available, cost-effective components and an advanced computer vision pipeline, the system achieves a 2.19 µm resolution, effectively resolving the G7.6 features from the USAF Resolving Power Test Target 1951. These results are comparable to those achieved by traditional optical microscopes under human cell counting, with the added benefits of portability and automation. Without forgetting the capabilities of the customization of the FOV (field of view) for our chip-sized microscope, thanks to the microdisplay and the vertical arrangement of our microscope, and due to its “multi-holographic” feature, we can “open” the FOV by losing the stitching area—which increases the noise in the sample but ensures a lower limit detection—or we can “close” the FOV increasing the stitching area—which ensures less noise and better reconstruction. This is a strong feature that enables the microscope’s adaptability to different measurement scenarios.

MicroVi was able to accurately detect and count yeast cells of *Saccharomyces cerevisiae.* Moreover, it was able to morphologically differentiate them from contaminant *Brettanomyces bruxellensis*, as well as bacteria like *Lactobacillus plantarum*, crucial for monitoring the fermentation process and quality control in wine production. Additionally, the system’s ability to perform holographic reconstructions and multi-holographic stitching expanded its applicability to more complex samples, such as red wine, vermouth and other wines. The exploration of alternative image processing techniques, such as projective homography and thin-plate splines for image stitching, as well as deep learning-based cell detection, offers promising directions for future development. These potential improvements, along with the high correlation between MicroVi’s automated cell counts and manual microscopy methods, underscore the system’s reliability and potential to standardize microbial analysis across different stages of winemaking.

By advancing both the hardware and software of microscope-on-a-chip solutions, this research opens new possibilities for their application in the wine industry and beyond, paving the way for more efficient and accurate microbial monitoring tools in a variety of industrial and biomedical fields.

## Figures and Tables

**Table 1:** A summary table of the physical properties of the optical mount for the microscope-on-a-chip device used in the MicroVi framework. (*) MicroVi can work with other µdisplays from the same manufacturer, which can change these values [12]. (**) Distance display—sample depends on each capture.

**Camera**	**Value**	**Units**
Max. resolution	3072 × 2048	pixels × pixels
Pitch	2.4	µm/pixel
**µDisplay**	**Value**	**Units**
Resolution	640 × 480	pixels × pixels
Pitch	4.0	µm/pixel
Wavelength (peak)	468 *	µm
Wavelength (width)	25 *	µm
**Mount**	**Value**	**Units**
Distance display—sample	500–1000 **	µm
Distance display—camera	10	mm

## Data Availability

The raw data supporting the conclusions of this article will be made available by the authors on request.
